# Polyhydroxyalkanoate production from rice straw hydrolysate obtained by alkaline pretreatment and enzymatic hydrolysis using *Bacillus* strains isolated from decomposing straw

**DOI:** 10.1186/s40643-021-00454-7

**Published:** 2021-10-08

**Authors:** Doan Van Thuoc, Nguyen Thi Chung, Rajni Hatti-Kaul

**Affiliations:** 1 Department of Biotechnology and Microbiology, Faculty of Biology, Hanoi National University of Education, 136 Xuan Thuy, Cau Giay, Hanoi, Vietnam; 2 Division of Biotechnology, Department of Chemistry, Center for Chemistry and Chemical Engineering, Lund University, P.O. Box 124, 221 00 Lund, Sweden

**Keywords:** *Bacillus* species, Polyhydroxyalkanoate, Rice straw, Mild alkaline pretreatment, Enzymatic hydrolysis

## Abstract

Rice straw is an important low-cost feedstock for bio-based economy. This report presents a study in which rice straw was used both as a source for isolation of bacteria producing the biodegradable polyester polyhydroxyalkanoate (PHA), as well as the carbon source for the production of the polymer by the isolated bacteria. Of the 100 bacterial isolates, seven were found to be positive for PHA production by Nile blue staining and were identified as *Bacillus* species by 16S rRNA gene sequence analysis. Three isolates showed 100% sequence identity to *B. cereus*, one to *B. paranthracis*, two with 99 and 100% identity to *B. anthracis*, while one was closely similar to *B. thuringiensis*. For use in PHA production, rice straw was subjected to mild alkaline pretreatment followed by enzymatic hydrolysis. Comparison of pretreatment by 2% sodium hydroxide, 2% calcium hydroxide and 20% aqueous ammonia, respectively, at different temperatures showed maximum weight loss with NaOH at 80 °C for 5 h, but ammonia for 15 h at 80 °C led to highest lignin removal of 63%. The ammonia-pretreated rice straw also led to highest release of total reducing sugar up to 92% on hydrolysis by a cocktail of cellulases and hemicellulases at 50 °C. Cultivation of the *Bacillus* isolates on the pretreated rice straw revealed highest PHA content of 59.3 and 46.4%, and PHA concentration of 2.96 and 2.51 g/L by *Bacillus cereus* VK92 and VK98, respectively.

**Supplementary Information:**

The online version contains supplementary material available at 10.1186/s40643-021-00454-7.

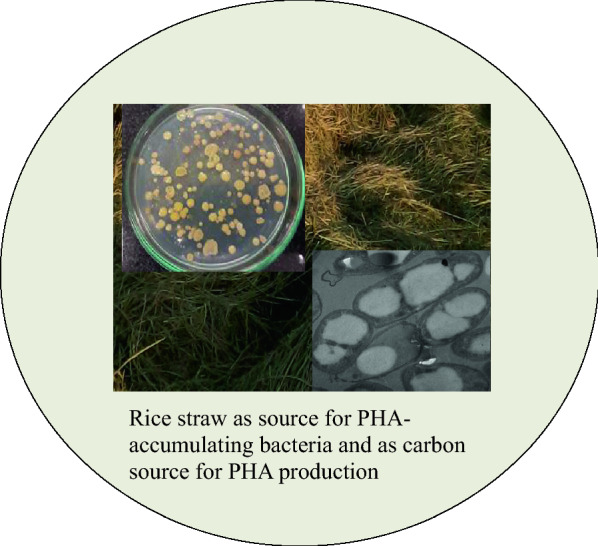

## Introduction

Rice straw is an obvious choice to be used as residual low-cost feedstock for the bio-based economy, especially in South- and South-East Asian countries that are the main producers of rice, the third most important grain crop in the world after wheat and corn (Binod et al. 2010). For every ton of rice grain, 700–1500 kg of rice straw is produced, and over 700 million tons of rice straw is produced worldwide (Bakker et al. 2013). In Vietnam, the total rice planted area is about 7.5 million ha, with a total of 40 million tons of rice and about 50 million tons of rice straw being produced annually (Diep et al. 2015). Some of the rice straw is collected for cooking, animal feed, roof covering or mushroom production. With improvement in the living conditions of farmers, such use of rice straw has become less common and 25–60% (depending on the region) is left for burning in the fields or considered as waste material (Bakker et al. 2013; Nguyen et al. 2016; Le et al. 2020;). Rice straw burning is estimated to release 11 tons of CO_2_-equivalent per hectare of land in addition to a large amount of NOx, a precursor to photochemical smog, and fine dust (Sarkar et al. 2012; Bakker et al. 2013). Valorization of rice straw would thus offset such emissions. Many studies claim that 25–35% of the straw may be available for biofuels and other products after leaving that is needed to conserve soil quality and for competitive uses (Bakker et al. 2013).

There have been many studies on the use of rice straw for the production of second-generation biofuel, and more recently even to other products like insulation materials, composites, biodegradable plastics and chemicals (Abraham et al. 2016; Bilo et al. 2018; Goodman 2020; Overturf et al. 2020). The biodegradable bio-based plastics that are increasingly attracting interest to replace the fossil-based plastics are the microbial polyesters—polyhydroxyalkanoates (PHAs) (Bedade et al. 2021; Naser et al. 2021; Yadav et al. 2021); their high rate of biodegradability even in marine environments lowers the risk of their accumulation as microplastics (Suzuki et al. 2021). As a major part of the production cost of PHA is attributed to the carbon source, use of rice straw could provide a potential low-cost alternative besides lowering the environmental impact caused by its burning (Obruca et al. 2015; Heng et al. 2016).

Rice straw contains over 70% carbohydrates in the form of cellulose and hemicellulose, access to which requires pretreatment, as for all lignocelluloses, for the removal of the lignin network (Binod et al. 2010; Sarkar et al. 2012). Unlike other agricultural residues, the straw also contains high ash content that poses a challenge in thermal processes leading to a high tendency for fouling the combustion systems. Various methods have been tested for pretreatment of rice straw (Hendriks and Zeeman 2009; Agbor et al. 2011; Tsegaye et al. 2019; 2020; Guo et al. 2020; Kurokochi and Sato 2020; Zang et al. 2020). Production of PHAs by *Bacillus firmus* and *Cupriavidus necator* using the hydrolysate of acid or alkali-pretreated rice straw with good yields has been reported (Sindhu et al. 2013; Ahn et al. 2015, 2016). Mild alkaline pretreatment has been widely applied on rice straw because of its simplicity, efficiency and relatively low cost. It increases the surface area by swelling the biomass particles and increasing carbohydrate accessibility to enzymes, while reducing the degree of polymerization and crystallinity of the cellulose (Tsegaye et al. 2019; Li et al. 2020). As very little of the carbohydrate is solubilized, most of it can be converted to sugars during the subsequent hydrolysis step (Hendriks and Zeeman 2009; Binod et al. 2010; Agbor et al. 2011; Ashoor and Sukumaran 2020).

The present report describes a study in which rice straw was used both as a source for isolation of PHA-producing bacteria, as well as the carbon source for the production of the polymer by the isolated bacteria. Decomposing rice straw was used for bacterial isolation, while dried straw pretreated with alkali followed by enzymatic hydrolysis was used as carbon source for PHA production. The effect of pretreatment with three different alkaline reagents (aqueous ammonia, sodium hydroxide and calcium hydroxide) at different temperatures on lignin removal and the sugar recovery from the enzymatic step was compared to find the best conditions for preparing the carbon source for PHA production.

## Materials and methods

### Rice straw

Rice straw (*Oryza sativa* L.) was collected from a field in the rural region of Dong Anh province in Vietnam, dried in air, ground and sieved. The particles that passed through a sieve with mesh size of 0.5 mm but not through a sieve with 0.2 mm mesh size were collected. Rice straw composition was analyzed using a standard analytical procedure (National Renewable Energy Laboratory (NREL), Golden, CO, USA) (Sluiter et al. 2008), and was determined to contain 43.1 ± 1.2% glucan, 17.7 ± 0.5% xylan, 3.0 ± 0.1% arabinan, 2.6 ± 0.1% galactan, and 12.9 ± 0.2% acid-insoluble lignin on a dry weight basis.

### Isolation of polyhydroxyalkanoate-producing bacteria

Decomposing rice straw was collected from the same field as above, ground, suspended in 0.9% NaCl solution, and the supernatant serially diluted, prior to spreading 100 µL of the diluted sample on a solid medium [meat–peptone–agar (MPA)] containing per liter: 5 g each of NaCl, meat extract, and peptone, and 20 g granulated agar. The plates were incubated at 35 °C for 48 h. Hundreds of colonies were picked and plated again on fresh MPA-agar medium. PHA-producing bacteria were then detected by Nile blue A staining method (Spiekermann et al. 1999), for which the bacterial isolates were grown on the modified MPA medium containing per liter: 5 g NaCl, 1 g meat extract, 1 g peptone, 20 g glucose, 20 g granulated agar, and Nile red (Sigma) (dissolved in dimethylsulfoxide) with a final concentration of 0.5 µg dye per mL of the medium. The agar plates were incubated at 35 °C for 2 days and then exposed to ultraviolet light (312 nm). The colonies with fluorescent bright orange staining were chosen for further studies.

### Phylogenetic characterization of the selected PHA-producing bacteria

The genomic DNA of the seven selected strains was extracted by Thermo Scientific GeneJET Genomic DNA Purification Kit according to the manufacturer’s recommendations. The 16S rRNA gene was amplified using the universal primers, 27F (5^’^-AGAGTTTGATCCTGGCTCAG-3^’^) and 1492R (5^’^-GGTTACCTTGTTACGCTT-3^’^). Sequencing of the amplified DNA fragment was performed at 1st Base (Singapore), and GenBank database was used to search for 16S rRNA gene similarities. Phylogenic analysis based on 16S rRNA gene was performed with the aid of MEGA X software (Kumar et al. 2018) using the Maximum Likelihood method and Tamura–Nei model (Tamura and Nei 1993). The almost complete sequences of the 16S rRNA gene of the bacterial strains were deposited in GenBank/EMBL/DDBJ databases and used in the analysis.

### Alkaline pretreatment of dried rice straw

Three different alkaline solutions (2% sodium hydroxide, 2% calcium hydroxide and 20% aqueous ammonia) were tested for the pretreatment of rice straw at solid:liquid ratio of 1:10. The mixtures of 10 g dry weight rice straw and 100 mL alkaline solution were placed in 250-mL glass bottles and incubated at different temperatures and time periods. Subsequently, the soaked rice straw was recovered by filtration, washed with clean water until neutral pH, and then dried at 105 °C for 24 h prior to enzymatic hydrolysis. Rice straw recovery was calculated based on the percent of amount of insoluble fraction recovered after pretreatment with respect to that before pretreatment.

### Enzymatic hydrolysis of the pretreated straw

Both pretreated and untreated rice straw samples were used as substrates for enzymatic hydrolysis. Three enzymes including Celluclast 1.5 L (129.3 mg protein/mL, 30.7 cellobiose units/mL and 63.8 filter paper units/mL), Novozyme 188 (102.2 mg protein/mL, 626.4 CBU/mL), and Pentopan Mono BG (2500 U/g) provided by Novozymes (Bagsvaerd, Denmark) were used, and the optimum conditions for rice straw hydrolysis were determined after several trials. Pretreated or untreated rice straw (1 g) was mixed with 25 mL of sodium acetate buffer (pH 5.0 containing 1% (v/v) of Celluclast 1.5L, 0.4% (v/v) of Novozyme 188 and 0.2% (w/v) of Pentopan in 100-mL glass bottles at 50 °C in a shaker incubator at 180 rpm for 40 h. Samples were withdrawn at different time intervals for monomeric sugar (glucose, xylose and arabinose) analysis.

### Polyhydroxyalkanoate production from rice straw hydrolysate using the bacterial isolates

The selected bacterial isolates were grown in 20 mL of liquid MPA medium in 100-mL Erlenmeyer flasks with rotary shaking at 180 rpm for 13 h. Subsequently, 1 mL of each culture was inoculated in 50 mL of modified MPA medium in 250-mL Erlenmeyer flasks. The medium contained per liter 5 g NaCl, 1 g meat extract, 1 g peptone, 20 g glucose or reducing sugars from hydrolysates, and the pH was initially adjusted to 7.0 using 0.05 M phosphate buffer. The cultures were incubated at 35 °C with rotary shaking at 180 rpm. Samples were withdrawn at 48 h of cultivation for determination of cell dry weight (CDW) and PHA content.

### Analytical methods

The surface characteristics of the untreated and pretreated rice straw were analyzed by scanning electron microscopy (SEM) (S-4800, Hitachi, Tokyo, Japan).

The PHA granules in the bacterial cells were observed by transmission electron microscopy (TEM) using JEM-1010 TEM (Jeol Korea Ltd., Seoul, South Korea).

The contents of sugars (glucose, xylose and arabinose) in the enzymatically hydrolyzed samples were determined using a HPLC system (Jasco, Tokyo, Japan) equipped with a reflective index detector (ERC, Taguchi, Japan). The sugars were separated on an Aminex HPX-87P column, using MilliQ water as mobile phase at a rate of 0.4 mL/min, and column temperature of 65 °C. The glucose concentration was used to calculate glucan-to-glucose conversion as follows:

$${\text{Glucan}}\;{\text{conversion}}\left( \% \right) = {\text{glucose}}\;{\text{liberated }}({\text{g}}) \times 0.{\text{9}} \times 100/{\text{initial}}\;{\text{cellulose}}(g)$$

Cell dry weight (CDW) was determined by centrifuging 3 mL of the culture samples at 4 000 *g* for 10 min in pre-weighed centrifuge tubes, the pellet was washed once with 3 mL distilled water, centrifuged and dried at 105 °C until constant weight was obtained. The tubes were weighed again to calculate the CDW.

PHA quantification was performed using a gas-chromatographic method (Huijberts et al. 1994). For this, about 10 mg of freeze-dried cells was mixed with 1 mL of chloroform and 1 mL of methanol solution containing 15% (v/v) sulphuric acid and 0.4% (w/v) benzoic acid. The mixture was incubated at 100 °C for 3 h to convert the constituents to their methyl esters. After cooling to room temperature, 0.5 mL of distilled water was added and the mixture was shaken for 30 s. The chloroform layer was transferred into a fresh tube and used for GC analysis to determine the PHA content. Sample volume of 2 μL was injected into the gas chromatography column (VARIAN, Factor Four Capillary Column, CP8907). The injection temperature was set at 250 °C, detector temperature at 240 °C, while the column temperature at 60 °C for the first 5 min and then increased at 3 °C/min until 120 °C was reached. Poly(3-hydroxybutyrate-co-3-hydroxyvalerate) (PHBV) containing 12% valerate (Sigma) was used as a standard for calibration.

Analyses of sugars, PHA and CDW were performed in triplicates.

PHA content (weight percent, wt%) was calculated as the percentage of the ratio of PHA concentration to CDW, while residual cell mass (RCM) was defined as the CDW minus PHA concentration (Lee et al. 2000). PHA yield (g/g) was calculated as concentration of the polymer divided by the amount of sugar used. PHA productivity (g/L/h) was calculated as PHA concentration divided by the cultivation time.

## Results and discussion

### Isolation and identification of PHA-producing bacteria from decomposing rice straw

**Fig. 1:**
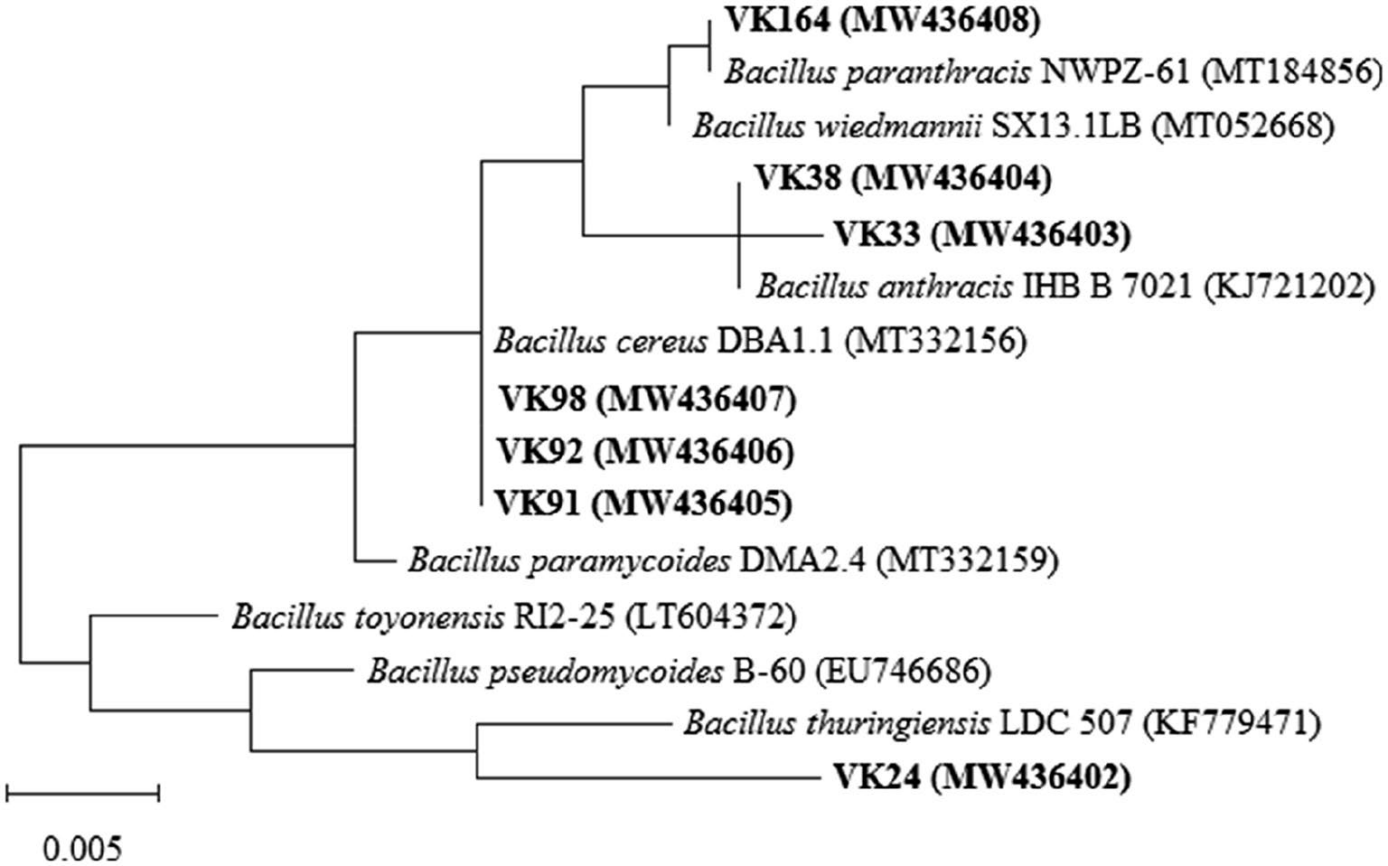
Maximum likelihood phylogenetic tree based on 16S rRNA gene sequences showing the relationships between the seven selected strains and other strains of the genus *Bacillus*. Bar 0.05 subtitutions per position

More than 100 bacterial colonies were isolated from decomposing rice straw, among which seven isolates were found to show significant PHA accumulation by Nile blue staining, and were used in this study. The phylogenetic characterization of the seven isolates based on their 16S rRNA gene sequences showed them to belong to genus *Bacillus* (Fig. 1). The sequence of strain VK24 showed a high level of similarity (98.2%) with that of *B. thuringiensis* LDC 507. Two strains VK33 and VK38 clustered together and showed the highest similarity of 99% and 100%, respectively, with *B. anthracis* IHB B 7021. Three other strains VK91, VK92 and VK98, also clustered together and showed similarity of 100% with *B. cereus* DBA1.1. Strain VK164 showed 100% sequence identity with *B. paranthracis* NWPZ-61 (100%). According to earlier reports, the bacterial populations degrading the straw under anoxic conditions comprised mainly *Clostridium* species with *Bacillus* species as one of the minor groups (Weber et al. 2001), while *Bacillus* species were predominant during composting of rice straw (Hefnawy et al. 2013). In fact, *Bacillus* species is used as an inoculant in rice straw composting for which they play an important role in degradation of cellulose and hemicellulose (Zhang et al. 2021).

PHA production by several *Bacillus* species such as *B. subtilis*, *B. firmus*, *B. cereus, B. thuringiensis* isolated from different ecosystems and using different carbon sources has been reported earlier (Singh et al. 2009; Sindhu et al. 2013; Gowda and Shivakumar 2014; Odeniyi and Adeola 2017; Mohandas et al. 2018; Ponnusamy et al. 2019; Mohammed et al. 2020). Synthesis of both homo- and copolymers has been reported depending on the substrate (Singh et al. 2009; Mohapatra et al. 2017).

### Mild alkaline pretreatment of rice straw and dry matter loss

Alkaline pretreatment of the dried rice straw was tested with 2% NaOH, 2% Ca(OH)_2_ and 20% aqueous NH_3_, respectively, at solid:liquid ratio of 1:10. These reagents have been used earlier for pretreatment of various biomass substrates including rice straw, rice hulls, switchgrass, corn stover, wood and bagasse (Chang et al. 1997, 1998; Park and Kim 2012; Sindhu et al. 2015; Heng et al. 2016; Kobkam et al. 2018; Tsegaye et al. 2019). Alkaline pretreatment solubilizes lignin by disrupting its structure and breaking the linkages with carbohydrate residues. It also removes acetyl and uronic acid substitutions on hemicellulose and helps to improve the accessibility of the polysaccharides to the hydrolytic enzymes (Carvalheiro et al, 2008; Bali et al. 2015; Kobkam et al. 2018; Tsegaye et al. 2019), but also resulting in their partial hydrolysis under strong alkaline conditions (Zhang and Cai 2008; Kobkam et al. 2018; Tsegaye et al. 2019).

In accordance with previous reports (Ko et al. 2009; Rodrigues et al. 2016; Sophonputtanaphoca et al. 2018), the amount of residual rice straw decreased with increase in temperature and time for the pretreatment; increase in temperature being a more effective parameter (Table 1). The highest straw recovery with 2% NaOH was 83-84% after 1 h treatment at 30–50 °C. Higher degree of solubilization was achieved during 1–5 h at 80 °C (Table 1), while removing 41–52% of lignin. In an earlier study, release of about 61% of hemicellulose and 36.2% of lignin was reported on treatment of chopped rice straw with 2% NaOH at solid:liquid ratio of 1:4, 85 °C for 1 h (Zhang and Cai 2008).

**Table 1:** Rice straw recovery after pretreatment with alkali at different incubation temperatures and times

2% (w/v) NaOH			2% (w/v) Ca(OH)_2_			20% aqueous ammonia		
Temperature (°C)	Time (h)	Rice straw recovery (%)	Temperature (°C)	Time (h)	Rice straw recovery (%)	Temperature (°C)	Time (h)	Rice straw recovery (%)
30	1	84.4 ± 1.3	30	12	90.7 ± 1.5	50	5	85.8 ± 1.7
	2.5	79.4 ± 1.5		24	89.4 ± 1.3		10	80.9 ± 1.4
	5	73.5 ± 1.5		36	89.4 ± 1.1		15	79.9 ± 1.3
	7.5	71.1 ± 1.4		48	88.0 ± 1.4	80	5	75.7 ± 1.2
	10	70.2 ± 1.2	80	12	88.7 ± 1.5		10	72.5 ± 1.5
50	1	83.3 ± 1.4		24	86.9 ± 1.6		15	70.5 ± 1.0
	2.5	71.6 ± 1.3		36	86.3 ± 1.2			
	5	67.4 ± 1.2		48	85.7 ± 1.1			
	7.5	64.4 ± 1.1	121	0.5	86.1 ± 1.7			
	10	63.4 ± 1.4		1	84.6 ± 1.9			
80	1	63.0 ± 1.0						
	2	61.5 ± 1.2						
	3	60.1 ± 1.3						
	4	59.1 ± 1.0						
	5	59.0 ± 1.2						

Pretreatment with 2% Ca(OH)_2_ resulted in rather high recovery of residual straw at all temperatures although slight decrease was seen with increasing time period; the lowest recovery (84.6%) being after 1 h treatment at 121 °C. Ca(OH)_2_ is a weaker alkali with low solubility in water and hence requires much longer treatment time and higher temperature, and moreover the calcium ions form calcium–lignin complex that hinder proper lignin solubilization (Rodrigues et al. 2016).

Aqueous NH_3_ is considered to be highly selective for lignin removal; it cleaves C–O–C bonds in lignin as well as ether and ester bonds in the lignin–carbohydrate complex (Binod et al. 2010), and has a significant swelling effect on lignocellulose. Residual straw recovery was intermediate to that obtained with NaOH and Ca(OH)_2_. Pretreatment with 20% aqueous NH_3_ for 15 h at 80 °C resulted in 29.5% of the weight loss of straw (Table 1), and 63% lignin removal (data not shown), which agrees well with the study by Park and Kim (2012) reporting removal of up to 66% lignin along with 22.9% hemicellulose and 11% cellulose from the rice straw treated with 15% aqueous NH_3_ (solid:liquid ratio of 1:10) at 60 °C for 24 h.

**Fig. 2:**
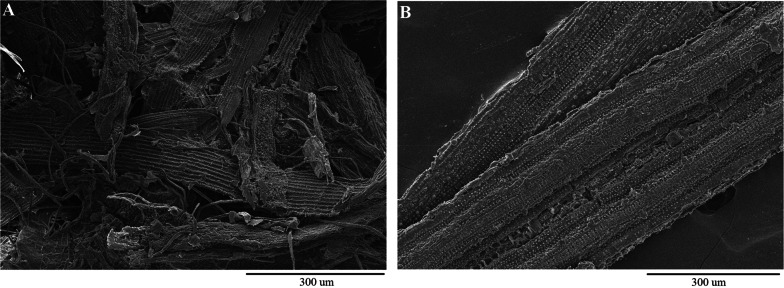
SEM micrographs of rice straw before (**A**) and after (B) pretreatment with 2% NaOH at 80 °C for 5 h

The alkaline-pretreated rice straw was much softer than the untreated one, indicating removal of large amounts of lignin and hemicellulose. As seen in Fig. 2, the rice straw pretreated with NaOH was distorted and separated from the initially connected structure, thus increasing the surface area (Fig. 2A) in comparison to the untreated straw with rigid, smooth and highly ordered fibrils (Fig. 2B).

### Enzymatic hydrolysis for releasing sugars from the polysaccharides

The effect of pretreatment with different alkaline reagents on the efficiency of enzymatic hydrolysis step was then evaluated (Additional files 1: Figures S1–S3). Both pretreated and untreated rice straw samples (1 g) were incubated in 4% w/v suspension at pH 5.0, 50 °C with an enzyme cocktail comprising 1% v/v cellulase preparation derived from *Trichoderma reesei* (Celluclast 1.5L), 0.4% v/v β-glucosidase from *Aspergillus niger* (Novozyme 188) and 0.2% w/v 1,4-β-xylanase from *Thermomyces lanuginosus* (Pentopan Mono BG). Glucose and xylose were the major products of hydrolysis besides a small amount of arabinose. Maximum yield of total reducing sugars from untreated rice straw was only 34.2%. In case of the straw pretreated with NaOH (Additional files 1: Figure S1), up to 72.2% of total reducing sugar was obtained for the sample subjected to pretreatment at 30 °C for 7.5 h while longer pretreatment time gave lower yield (Additional files 1: Figure S1A). Increasing the pretreatment temperature to 50 °C (2.5 or 5 h) and 80 °C (3 h) resulted in a slight increase in the sugar yield to about 74% (Additional files 1: Figure S1B) and 77% (Additional files 1: Figure S1C), respectively. In case of Ca(OH)_2_-pretreated rice straw, the highest sugar yield of 60–66% was obtained for the samples pretreated at 30 °C for 48 h (Additional file 1: Figure S2A), or 80 °C for 24 h (Additional files 1: Figure S2B), while significantly lower yield was noted when the pretreatment was performed at 121 °C (Additional files 1: Figure S2C). Pretreatment with aqueous ammonia gave the highest sugar yield ranging between 77 and 87% upon enzymatic hydrolysis (Additional file 1: Figure S3A, B), the highest yield being obtained for the straw pretreated at 80 °C for 15 h (Additional file 1: Figure S3B). However, a much longer time (30 h) for enzymatic treatment was needed to obtain the maximal sugar release as compared to that for the samples treated with Ca(OH)_2_ and NaOH which took about 15 h for the maximal release.

Table 2 shows that the glucan conversion efficiency obtained in this study was in the same range as that in previous studies. A few studies have reported slightly higher yields for the straw pretreated either at a higher temperature, longer time or lower solid loading (Ko et al. 2009; Kim and Han 2012; Kim et al. 2014; Tsegaye et al. 2019).

**Table 2:** Comparison of the glucan-to-glucose conversion of cellulose in rice straw treated with different pretreatment methods followed by enzymatic hydrolysis

Pretreatment method	Solid loading (g/L)	Hydrolysis time (h)	Glucan conversion (%)	Reference
20% aq. ammonia, 80 °C, 10 h	100	30	92	This study
21% aq. ammonia, 69 °C, 10 h	167	96	88.4	Ko et al. (2009)
20% aq. ammonia, 80 °C, 12 h	100	72	94.4	Kim et al. (2014)
7% aq. ammonia, 80 °C, 12 h	100	72	91.2	Kim et al. (2014)
2% NaOH, 80 °C, 3 h	100	15	81.9	This study
2.96% NaOH, 81.79 °C, 56.66 min	100	72	78.7	Kim and Han (2012)
4% NaOH, 25 °C, 12 h and 121 °C, 1 h	80	96	81.2	Takano and Hoshino (2018)
5% NaOH, 100 °C, 3 h	30	24	84.33	Sophonputtanaphoca et al. (2018)
7% NaOH, 80 °C, 4 h	100	48	88.27	Tsegaye et al. (2019)
2% Ca(OH)_2_, 80 °C, 24 h	100	15	55.6	This study
10% Ca(OH)_2_, 95 °C, 3 h	100	72	48.5	Cheng et al. (2010)
15% Ca(OH)_2_, 25 °C, 72 h	50	72	58.4	Gu et al. (2015)
1% H_2_SO_4_, 160 °C, 0.5 h	50	72	57	Lee et al. (2015)
3.65% HCl, 4.9% H_2_SO_4_ or 3.27% H_3_PO_4_, 25 °C, 4 h and 121 °C, 1 h	100	120	72,2%	Jampatesh et al. (2019)
1% H_2_SO_4_, 1 h and steam explosion, 180 °C, 10 min residence time	50	72	81.1%	Semwal et al. (2019)

### PHA production from the rice straw hydrolysate

**Fig. 3:**
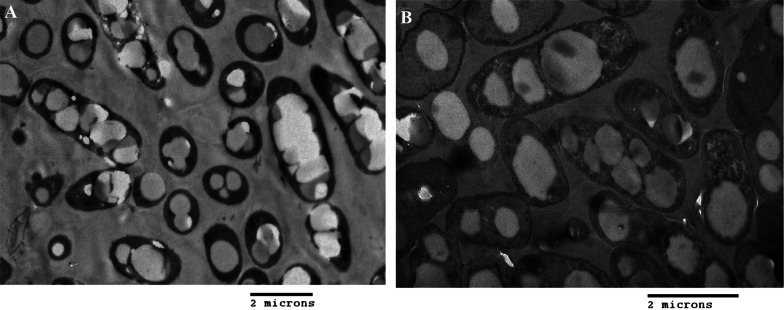
TEM micrographs of PHA granules accumulated by strain VK98 on: **A** glucose-based culture medium and **B** rice straw hydrolysate-containing medium, respectively

The seven bacterial isolates were cultured in the medium containing 20 g/L glucose or 20 g/L of reducing sugars obtained by saccharification of the ammonia-pretreated rice straw followed by enzymatic hydrolysis at pH 5.0 and re-adjusting the pH to 7.0. The CDW, PHA content and concentration obtained after 48 h of cultivation are summarized in Tables 3 and 4. All the seven strains grew well in the glucose medium with CDW ranging from 2.2 to 3.32 g/L, and the PHA content ranged between 42 and 73 wt% of the cell dry weight, the highest value being obtained for the strain *B. cereus* VK98 (Table 3 and Fig. 3A). The final cell mass obtained was generally lower in the straw hydrolysate medium, the only exceptions being the strains *B. cereus* VK92 and VK98 that gave CDWs of 5 g/L and 5.42 g/L, respectively (Additional file 1: Figures S4, S5). The PHA content was also highest in the *B. cereus* strains, 59.3 wt% in VK92, followed by 46.4 wt% in VK98 (Additional file 1: Figures S4, S5) and 43.8 wt% in VK 91 (Table 4). Figure 3A and B shows the TEM micrographs at 2 μm resolution of the PHA granules accumulated by the strain VK98 grown with glucose and straw hydrolysate, respectively. The polymer was primarily polyhydroxybutyrate (PHB) with traces of hydroxyvalerate. Glucose was fully consumed, but the bacteria did not utilize xylose. *B. anthracis* VK33, *B. anthracis* VK38, *B. paranthracis* VK164 exhibited PHA content of less than 10 wt%, while no PHA was detected in the cells of *Bacillus* sp. VK24. This suggests *B. cereus* VK92 and VK98 are resistant to the inhibitory compounds formed during the pretreatment and autoclaving. These include weak acids such as acetic acid, glycolic acid, formic acid and levulinic acids, and phenolic compounds, e.g., coumaric acid, syringaldehyde, 4-hydroxybenzaldehyde, and vanillin (Jönsson et al. 2013; van der Pol et al. 2016). VK92 and VK98 also exhibited higher final pH values (Table 4), which may suggest that these isolates are able to utilize some of the acids and transform them to other metabolic products or perhaps even to PHA with different monomer composition (Vu et al. 2021; Ahn et al. 2016). This needs, however, to be further investigated.

**Table 3:** Growth and PHA accumulation in seven *Bacillus* species in the medium containing 20 g/L glucose

Strains	Initial pH	Final pH	CDW (g/L)	PHA content (wt%)	PHA conc (g/L)	RCM (g/L)
*Bacillus* sp. VK24	7.0	5.3	2.20 ± 0.05	42.0 ± 0,7	0.92 ± 0.01	1.28 ± 0.04
*B. anthracis* VK33	7.0	5.2	2.33 ± 0.05	47.2 ± 1.4	1.10 ± 0.05	1.23 ± 0.01
*B. anthracis* VK38	7.0	5.2	3.02 ± 0.07	63.8 ± 0.9	1.93 ± 0.07	1.09 ± 0.01
*B. cereus* VK91	7.0	5.2	3.23 ± 0.09	66.9 ± 0.3	2.16 ± 0.05	1.07 ± 0.04
*B. cereus* VK92	7.0	5.2	3.15 ± 0.16	59.9 ± 0.8	1.89 ± 0.12	1.26 ± 0.04
*B. cereus* VK98	7.0	5.3	3.32 ± 0.02	73.2 ± 0.1	2.43 ± 0.02	0.89 ± 0.01
*B. paranthracis* VK164	7.0	5.2	2.47 ± 0.05	56.5 ± 2.5	1.39 ± 0.11	1.07 ± 0.07

**Table 4:** Growth and PHA accumulation by seven isolated *Bacillus* species in the medium containing 20 g/L reducing sugars produced by saccharification of aqueous ammonia-pretreated rice straw

Strains	Initial pH	Final pH	CDW (g/L)	PHA content (wt%)	PHA conc (g/L)	RCM (g/L)
*Bacillus* sp. VK24	7.0	5.5	1.60 ± 0.05	0	0	1.60 ± 0.05
*B. anthracis* VK33	7.0	5.5	2.28 ± 0.07	3.3 ± 0.1	0	2.20 ± 0.07
*B. anthracis* VK38	7.0	5.4	2.85 ± 0.02	5.9 ± 0.3	0.17 ± 0.01	2.68 ± 0.03
*B. cereus* VK91	7.0	5.6	2.80 ± 0.09	43.8 ± 1.3	1.23 ± 0.01	1.57 ± 0.09
*B. cereus* VK92	7.0	8.0	5.00 ± 0.19	59.3 ± 0.7	2.96 ± 0.15	2.04 ± 0.04
*B. cereus* VK98	7.0	7.1	5.42 ± 0.12	46.4 ± 2.7	2.51 ± 0.09	2.90 ± 0.21
*B. paranthracis* VK164	7.0	5.6	2.67 ± 0.19	9.1 ± 0.2	0.24 ± 0.01	2.43 ± 0.18

Comparison of the results obtained in this study with earlier reports on PHA production from rice straw hydrolysate clearly shows the production parameters to be highly comparable (Table 5). Majority of the other studies have utilized acid-hydrolyzed rice straw. The highest accumulation of PHB (89% w/w) was reported in *Bacillus firmus* grown on pentose-rich hydrolysate with 0.75% xylose but at a much lower cell mass, hence yielding low PHB concentration (1.7 g/L) (Sindhu et al. 2013). On the other hand, *Ralstonia eutropha* (or *C. necator*), the most commonly used bacteria for PHA production, yielded the highest amount of PHB (9.88 g/L, 70.1% w/w) from the rice straw pretreated with NaOH followed by two-stage enzymatic hydrolysis (Saratale and Oh 2015) instead of the acid-hydrolyzed straw (Ahn et al. 2015, 2016). The high polymer concentration obtained in *R. eutropha* was primarily due to the very high concentration of the cell mass (15.5 g/L) but also high PHA accumulation in the cells.

**Table 5:** Comparison of PHA production by different bacterial strains from rice straw hydrolysates

Strains	Pretreatment—hydrolysis method	CDW (g/L)	PHA content (wt%)	PHA conc (g/L)	Yield (g/g)	PHA (g/L/h)	Reference
*Bacillus cereus* VK92	20% NH_3_, 80 °C, 10 h—Enzymatic, 50 °C, 40 h	5.00	59.3	2.96	0.15	0.062	This study
*B. cereus* VK98	20% NH_3_, 80 °C –Enzymatic, 50 °C, 40 h	5.42	46.4	2.51	0.13	0.052	This study
*Bacillus firmus* NII 0830^a^	2% H_2_SO_4_, 121 °C, 1 h	1.90	89.0	1.68	0.22	0.019	Sindhu et al. (2013)
*Cupriavidus necator* ATCC 17697	6% H_2_SO_4_,121 °C, 1 h	1.71	21.0	0.36	0.13	0.03	Ahn et al. (2015)
*C. necator* ATCC 17697	2% H_2_SO_4_, 121 °C, 1 h	1.59	53.0	0.84	0.13	0.035	Ahn et al. (2016)
*Ralstonia eutropha* (*C. necator*) ATCC 17697	2% NaOH, 121 °C, 30 min—Enzymatic in two stages, 50 °C and 24 h for each stage	15.5	70.1	9.88	0.49	0.206	Saratale and Oh (2015)
*Bacillus megaterium* B-10	0.5% H_2_SO_4_, 121 °C, 40 min	4.67	32.0	1.50	0.08	0.042	Li et al. (2021)

^a^PHB analysis using spectrophotometric method

Moderate PHA yield of 0.15 g/g was obtained with respect to the sugar consumed in the *B. cereus* isolates (Table 5), which was equivalent to 59.5 g PHA per kg rice straw in case of *B. cereus* VK92, considering also the losses during the pretreatment. It is possible to further improve the PHA content and productivity in the isolates by medium optimization, e.g., with respect to carbon:nitrogen ratio, phosphate level, aeration, etc., and by using fed-batch mode of cultivation under controlled conditions (Singh et al. 2021). Moreover, strategies to further improve the resource- and cost-efficiency need to be developed when utilizing rice straw as feedstock, e.g., by transformation of other components to other value added products.

## Conclusions

This study shows the potential of utilizing the bacteria, involved in degradation of rice straw, as hosts for PHA production from reducing sugars liberated by pretreatment and hydrolysis of the rice straw lignocelluloses. Aqueous ammonia soaking pretreatment was found to be an efficient method for lignin removal from rice straw, providing suitable substrate for enzymatic digestibility of the polysaccharides. Further improvements in PHA content and productivity are possible by optimization of the culture medium and mode of cultivation. The pretreatment and the enzymatic hydrolysis, especially the latter, are the most cost-determining steps for PHA production from rice straw. In order to reduce these costs, it would be interesting to investigate PHA production in association with the *Bacillus* species that are involved in composting of rice straw through their polysaccharide-degrading activities (Hefnawy et al. 2013; Zhang et al. 2021), and even to consider developing a biorefinery by co-production of other chemicals and materials from the feedstock.

## Supplementary Information

**Additional file 1: Fig S1**. Yield of reducing sugars obtained after enzymatic hydrolysis of NaOH pretreated rice straw at (A) 30°C, (B) 50°C, and (C) 80°C, respectively, for different time periods. The enzymatic treatment was performed at 50°C. **Fig S2**. Yield of reducing sugars obtained during enzymatic hydrolysis of rice straw pretreated with Ca(OH)2 for different time periods at (A) 30°C, (B) 80°C, and (C) 121°C, respectively. The enzymatic treatment was performed at 50°C. **Fig S3**. Yield of reducing sugars obtained after enzymatic hydrolysis of rice straw pretreated with aqueous ammonia for different incubation times at (A) 50°C, and (B) 80°C, respectively. The enzymatic treatment was performed at 50°C. **Fig S4**. Cell growth and PHA accumulation by the strain B. cereus VK92 in culture media using (A) glucose and (B) rice straw hydrolysate, as carbon source at 35°C. **Fig S5**. Cell growth and PHA accumulation by strain B. cereus VK98 in culture media using (A) glucose and (B) rice straw hydrolysate, as carbon source at 35°C.

## Data Availability

The raw data supporting the conclusion of this article will be made available by the authors without undue reservation.
