# Correction: Polyhydroxyalkanoate production from rice straw hydrolysate obtained by alkaline pretreatment and enzymatic hydrolysis using *Bacillus* strains isolated from decomposing straw

**DOI:** 10.1186/s40643-024-00804-1

**Published:** 2024-09-23

**Authors:** Doan Van Thuoc, Nguyen Thi Chung, Rajni Hatti-Kaul

**Affiliations:** 1 Department of Biotechnology and Microbiology, Faculty of Biology, Hanoi National University of Education, 136 Xuan Thuy, Cau Giay, Hanoi, Vietnam; 2 Division of Biotechnology, Department of Chemistry, Center for Chemistry and Chemical Engineering, Lund University, P.O. Box 124, 221 00 Lund, Sweden

**Correction: Bioresources and Bioprocessing (2021) 8:98**

10.1186/s40643-021-00454-7

The authors have recently identified an error concerning Fig. 3A. The transmission electron micrograph shown in the figure is mistakenly the one (also Fig. 3A) from a paper published in 2012 by the first author: Doan Van Thuoc et al. (2012) Polyester production by halophilic and halotolerant bacterial strains obtained from mangrove soil samples located in Northern Vietnam. *Microbiology Open* 2012. 10.1002/mbo3.44.

Fig. 3 with the correct Fig. 3A is now included:

**Fig. 3:**
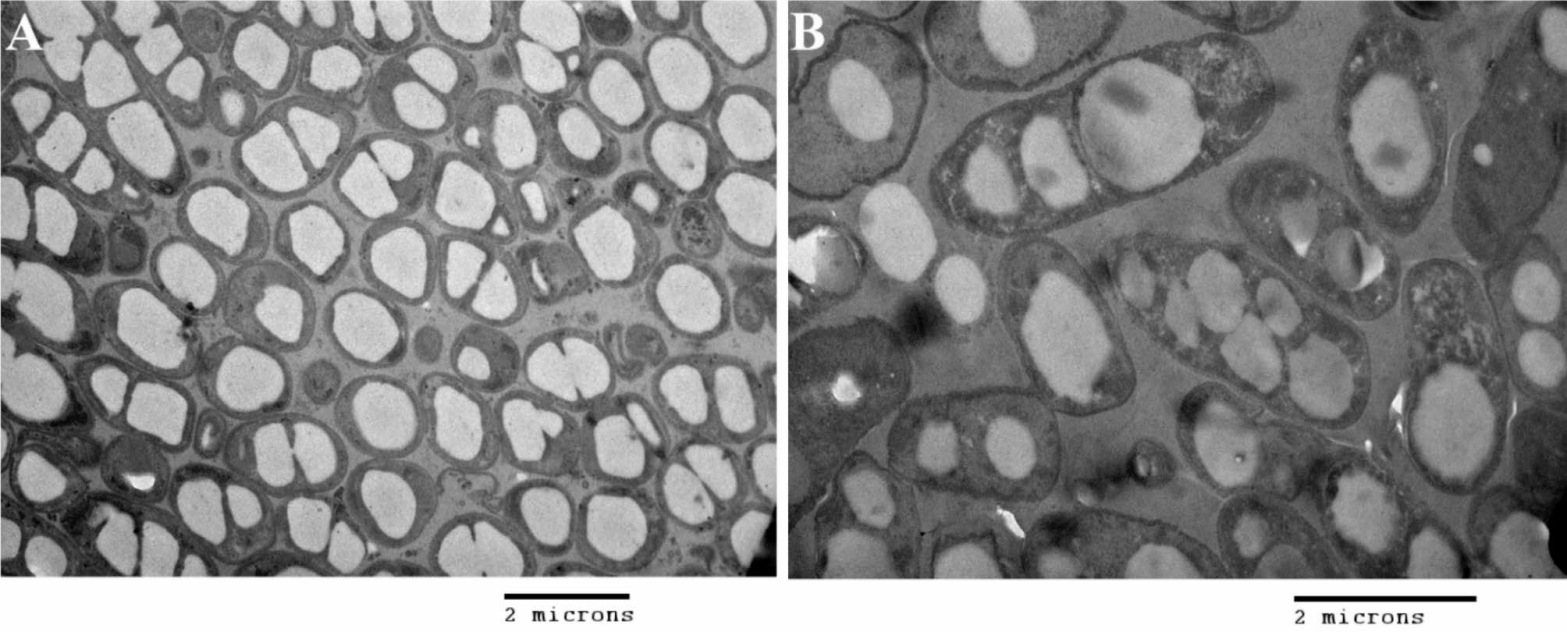
TEM micrographs of PHA granules accumulated by strain VK98 on: **A** glucose-based culture medium and **B** rice straw hydrolysate-containing medium, respectively

