# Supplementary material for: Polyhydroxyalkanoate production from rice straw hydrolysate obtained by alkaline pretreatment and enzymatic hydrolysis using Bacillus strains isolated from decomposing straw
Source: Bioresour Bioprocess. 2021 Oct 8;8(1):98. doi: 10.1186/s40643-021-00454-7 (PMC10992000; doi:10.1186/s40643-021-00454-7)
Supplement: Supplementary file 1 — Additional file 1: Fig S1. Yield of reducing sugars obtained after enzymatic hydrolysis of NaOH pretreated rice straw at (A) 30°C, (B) 50°C, and (C) 80°C, respectively, for different time periods. The enzymatic treatment was performed at 50°C. Fig S2. Yield of reducing sugars obtained during enzymatic hydrolysis of rice straw pretreated with Ca(OH)2 for different time periods at (A) 30°C, (B) 80°C, and (C) 121°C, respectively. The enzymatic treatment was performed at 50°C. Fig S3. Yield of reducing sugars obtained after enzymatic hydrolysis of rice straw pretreated with aqueous ammonia for different incubation times at (A) 50°C, and (B) 80°C, respectively. The enzymatic treatment was performed at 50°C. Fig S4. Cell growth and PHA accumulation by the strain B. cereus VK92 in culture media using (A) glucose and (B) rice straw hydrolysate, as carbon source at 35°C. Fig S5. Cell growth and PHA accumulation by strain B. cereus VK98 in culture media using (A) glucose and (B) rice straw hydrolysate, as carbon source at 35°C. [file 40643_2021_454_MOESM1_ESM.docx]

Supplementary file

**Polyhydroxyalkanoate production from rice straw hydrolysate obtained by alkaline pretreatment and enzymatic hydrolysis using *Bacillus* strains isolated from decomposing straw**

**Doan Van Thuoc^1^, Nguyen Thi Chung^1^, Rajni Hatti-Kaul^2*^**

^1^Department of Biotechnology and Microbiology, Faculty of Biology, Hanoi National University of Education, 136 Xuan Thuy, Cau Giay, Hanoi, Vietnam

^2^Division of Biotechnology, Department of Chemistry, Center for Chemistry and Chemical Engineering, Lund University, P.O. Box 124, SE-221 00 Lund, Sweden

*^*^*Corresponding author

Tel: +46-46-222 4840

Telefax: +46-46-222 4713

E-mail: Rajni.Hatti-Kaul@biotek.lu.se

**Fig S1.** Yield of reducing sugars obtained after enzymatic hydrolysis of NaOH pretreated rice straw at (A) 30°C, (B) 50°C, and (C) 80°C, respectively, for different time periods. The enzymatic treatment was performed at 50°C.

**Fig S2.** Yield of reducing sugars obtained during enzymatic hydrolysis of rice straw pretreated with Ca(OH)_2_ for different time periods at (A) 30°C, (B) 80°C, and (C) 121°C, respectively. The enzymatic treatment was performed at 50°C.

**Fig S3.** Yield of reducing sugars obtained after enzymatic hydrolysis of rice straw pretreated with aqueous ammonia for different incubation times at (A) 50°C, and (B) 80°C, respectively. The enzymatic treatment was performed at 50°C.

**Fig S4.** Cell growth and PHA accumulation by the strain *B. cereus* VK92 in culture media using (A) glucose and (B) rice straw hydrolysate, as carbon source at 35°C.

**Fig S5.** Cell growth and PHA accumulation by strain *B. cereus* VK98 in culture media using (A) glucose and (B) rice straw hydrolysate, as carbon source at 35°C.
